# 

**Published:** 1872-01-15

**Authors:** 


Money Receipts to Jan. 20th, 1872.—Dr.'s C. T. Hunter, \$5.00; E. R. Willard, \$3.00; C. H. Allen, \$3.00; W. W. McMann, \$3.00; T. Griffin, \$3.00; V. L. Hurlbut, \$3.00; M. Reece, \$3.00; L. Humphreys, \$3.00; J. B. Meigs, \$3.00; R. W. Bower, \$3.00; Doepp, \$3.00; J. J. Tyke, \$6.00; Thos. Hamill, \$3.00; D. Prince, \$3.00; W. M. Helm, \$5.00; H. Nance, \$3.00; H. W. Alexander, \$3.00; A. Given, \$3.00; N. Senn, \$3.00; W. H. Buchtel, \$3,00; A. C. Carr, \$6.00.

THE

### MEDICAL EXAMINER.

A SEMI-MONTHLY JOURNAL,

DEVOTED TO THE

Educational, Scientific & Practical Interests

OF

*THE MEDICAL PROFESSION.*

EDITED BY

N. S. DAVIS, M.D., and F. H. DAVIS, M.D.

### PROSPECTUS

FOR 1872.

With the commencement of Volume XIII, January, 1872, THE EXAMINER will be issued in a new and enlarged form, semi-monthly, instead of monthly, as heretofore.

ITS CONTENTS

Will be of such a character as will directly aid the Physician in the daily practical duties of his profession.

A LARGE LIST OF CONTRIBUTORS,

Including many of the most Eminent Physicians and Surgeons in the West insures to the EXAMINER an abundant supply of original articles.

THE HOSPITAL REPORTS

Will include Abstracts of all the Principal Clinics of the Mercy Hospital, the County Hospital and the Free Dispensaries, while

THE SELECT DEPARTMENT

Will contain a Careful Digest of the Professional and Scientific News and Literature, BOTH AT HOME AND ABROAD.

*Yearly Subscriptions, - $3.00*

*In Advance; Single Numbers, 15 Cents.*

☞ Liberal Commissions offered to those who will interest themselves in obtaining New Subscribers for the EXAMINER.

☞ For a Club of FOUR NEW SUBSCRIBERS, and $12.00, we will send an extra copy of the EXAMINER for one year.

ADDRESS,

**Publishers Medical Examiner,**

*797 Wabash Avenue,*

*CHICAGO.*

